# Development of a Customizable Programme for Improving Interprofessional Team Meetings: An Action Research Approach

**DOI:** 10.5334/ijic.3076

**Published:** 2018-01-25

**Authors:** Jerôme Jean Jacques van Dongen, Marloes Amantia van Bokhoven, Wilhelmus Nicolaas Marie Goossens, Ramon Daniëls, Trudy van der Weijden, Anna Beurskens

**Affiliations:** 1 Research Centre for Autonomy and Participation for People with Chronic Illnesses, Faculty of Health, Zuyd University of Applied Sciences, 6400 AN, Heerlen, NL; 2 Department of Family Medicine, Care and Public Health Research Institute (CAPHRI), Maastricht University, 6200 MD Maastricht, Maastricht, NL

**Keywords:** interprofessional collaboration, interprofessional team meetings, integrated care, qualitative research, chronic diseases, action research

## Abstract

**Introduction:**

Interprofessional teamwork is increasingly necessary in primary care to meet the needs of people with complex care demands. Needs assessment shows that this requires efficient interprofessional team meetings, focusing on patients’ personal goals. The aim of this study was to develop a programme to improve the efficiency and patient-centredness of such meetings.

**Methods:**

Action research approach: a first draft of the programme was developed, and iteratively used and evaluated by three primary care teams. Data were collected using observations, interviews and a focus group, and analysed using directed content analysis.

**Results:**

The final programme comprises a framework to reflect on team functioning, and training activities supplemented by a toolbox. Training is intended for the chairperson and a co-chair, and aims at organizing and structuring meetings, and enhancing patient-centredness. Our findings emphasize the essential role of the team’s chairperson, who, in addition to technically structuring meetings, should act as a change agent guiding team development.

**Conclusion:**

Findings show that the programme should be customizable to each individual team’s context and participants’ learning objectives. Becoming acquainted with new structures can be considered a growth process, in which teams have to find their way, with the chairperson as change agent.

## Background

Today, chronic diseases are responsible for 60% of the global disease burden. Due to increased life expectancy, it is expected to rise to, 80% by the year 2020 [1]. Chronically ill patients often suffer from multiple chronic conditions, and use health services more often than other patients [2]. The traditional definition of the World Health Organisation (WHO), which defines health as complete well-being no longer applies to these patients. Current definitions tend to view health from a multidimensional perspective and focus on resilience, in the face of social, physical and emotional challenges [3]. In contrast to the traditional definition by the WHO, this new concept is more holistic, including multiple domains. The key to a better understanding of these domains and patients’ individual needs appears to be patient-centred care, which is associated with improved health status and efficiency of care [4]. In patient-centred care, the patient is considered as a whole person with biological, psychological and social needs [5].

Given their focus on structures and processes that position patients at the center, integrated care systems appear to offer an ideal platform to optimise patient-centred care [6]. Contributions of professionals from different disciplines need to be integrated into joint care plans [5,7,8,9,10,11]. In the Dutch primary care setting, interprofessional collaboration is often implemented through periodic interprofessional team (IPT) meetings. During these meetings professionals aim to develop integrated patient-centred care plans and coordinate the care process. Besides, interprofessional team meetings are a way to foster informal learning among professionals [12].

Interprofessional primary care team meetings appear to vary considerably in format and content. An average team may comprise family physicians, practice nurses, occupational therapists, physical therapists, and district nurses [13].

Conducting successful IPT meetings is complex as it is influenced by many factors [9,13,14,15]. A structure for team meetings, preparation of the meetings, division of roles and shared values have been reported to be important aspects of successful and efficient team meetings [13]. In practice, teamwork is often inefficient and time consuming, due to lack of a clear structure, depth of discussion and lack clear agreements [13,16]. Professionals consider lack of time to be a serious barrier to team functioning [14]. Moreover, observations have shown that IPT meetings are still dominated by the professional instead of the individual patients’ perspective and often lack a leader who can guide team development through stimulating team reflection [13].

Consequently, there is a need for effective interventions to improve IPT meetings [16]. A needs assessment encompassing various qualitative studies and a scoping review [13,14,15,17] showed that current practice could benefit from improvements in structure, patient-centredness and leadership. The aim of the present study is to systematically develop the format and content of a programme for improving interprofessional team meetings focusing on these prioritized topics.

## Methods

### Design

An action research design was used to develop the programme. The rationale for using action research was to develop a programme in an iterative approach by learning through action, and ensuring the involvement of intended users (health care professionals). Action research is usually conducted in four phases (**Box 1**) [18].

**Box 1: Phases of action research**

1. **Plan** (development of the draft programme)
2. **Act and observe** (testing the draft programme)
3. **Reflect** (evaluation of the usability in context)
4. **Revised plan** (final programme)

#### Phase 1: Plan

**Figure 1:**
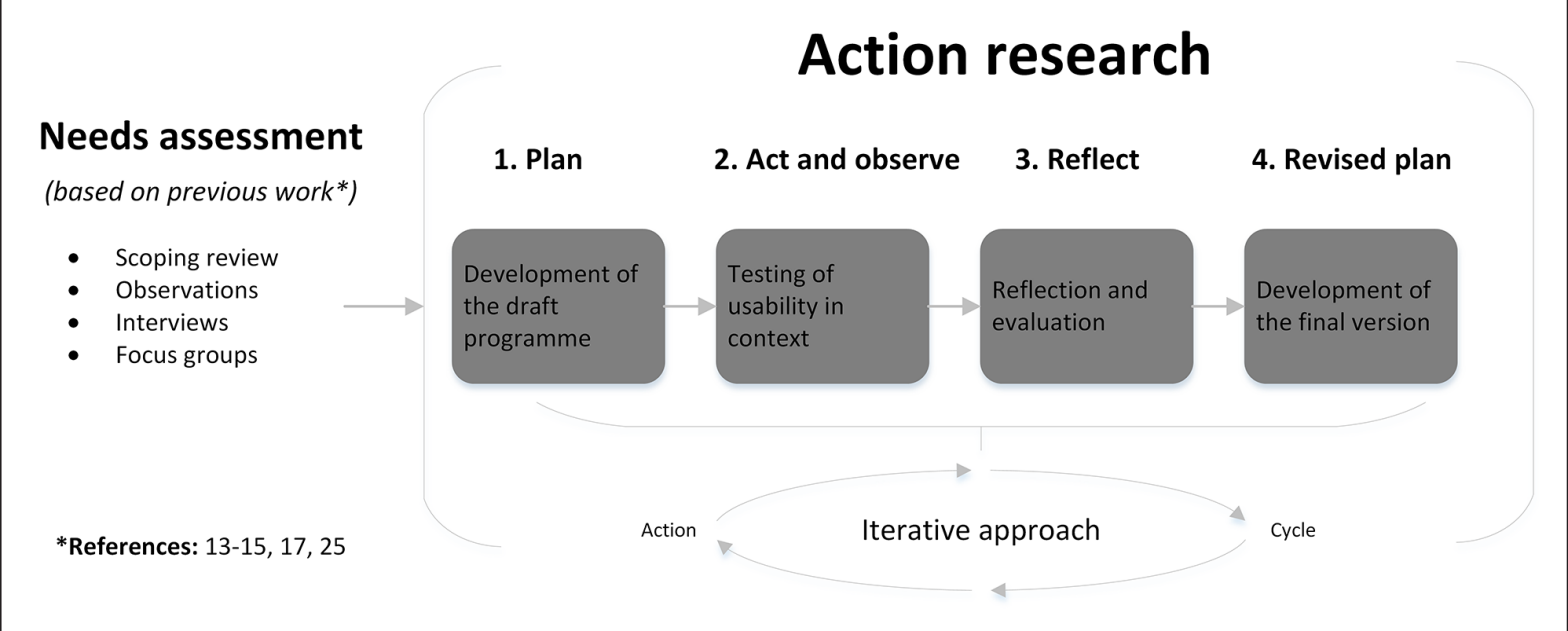
Programme development process.

**Figure 2:**
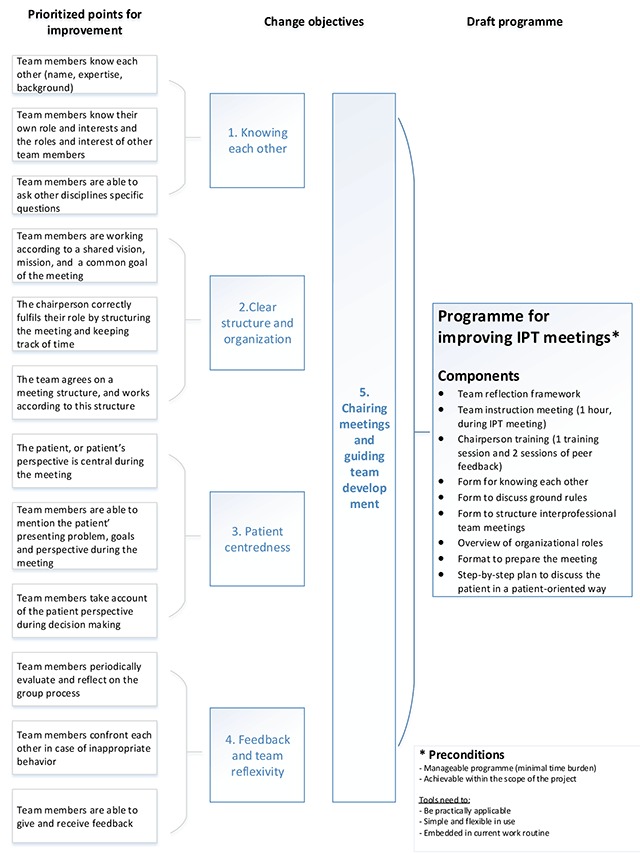
Development of the draft programme.

In the first phase a development team developed the draft programme. Figure 1 shows the programme’s development process. The input for the development team consisted of a list of topics for improvement based on previous work (compiled during a needs assessment [13,14,15,17]), which were prioritized during a consensus meeting by an expert panel including eight experts and professionals, and one patient representative. This resulted in 12 points for improvement, see Figure 2, left column. Subsequently, based on these points for improvement five main change objectives were derived by the development team: (1) knowing each other personally, (2) clear structure and organization, (3) patient-centredness, (4) feedback and team reflexivity, and 5) chairing meetings and guiding team development (Figure 2, middle column). The development team gathered five times during the period May–June 2014. Firstly, the team studied the change objectives as derived from the needs assessment. Secondly, the team defined the preconditions of the programme (Figure 2). They then started a preliminary exploration of ideas, in which ideas were not immediately rejected based on their feasibility, and thinking outside the box was encouraged by using pratical working methods. During the subsequent meetings, preliminary ideas were converted into actual drafts.

#### Phase 2 and 3: Act and observe and reflect

The second and third phases involved evaluating the programmes usability in context through four iterative action research cycles. Each cycle comprised a period of acting and observing, reflecting and revising (Figure 1). In between the cycles, participating teams had the opportunity to experiment and adjust the materials.

#### Phase 4: Revised plan

In the fourth phase, the development team revised and adjusted the draft programme based on the findings of the previous phases, resulting in a set of criteria and the final programme. The programme was developed over a 6-month period (Jan–Jun 2015).

### Participants

#### Development team (phases 1 and 4)

In the first phase, participants for the development team were recruited by means of purposive sampling using the researchers’ network, including at least one researcher, one health care professional, one expert in group dynamics, and one creative designer. Eventually five professionals took part in the development team (two researchers, a practice nurse, a creative designer, and an expert in group dynamics). Oral informed consent was obtained from all participants.

#### Participating teams (phases 2 and 3)

In order to test and evaluate the draft programme within the intended context, three interprofessional primary care teams were recruited by means of purposive sampling using the researchers’ network. Teams were included in the study if they periodically conducted IPT meetings including three or more health care professionals from different disciplines. In addition, the teams’ chairpersons should be willing to participate in a training programme. The meetings were part of the usual care process, and not initiated specifically for this study. Team members received oral information, and a letter with information about the content of the study and confidentiality of the data. Oral informed consent was obtained from all participants. The characteristics of the three participating teams are presented in Table 1.

**Table 1:** Characteristics of the participating interprofessional primary care team meetings.

Team	Duration in minutes	Frequency of team meetings	Average number of participants	Disciplines

1	90	Once every two months	7	Family physician, practice nurse, physical therapist, occupational therapist, pharmacist
2	60	Once a month	14	Family physician, practice nurse, physical therapist, occupational therapist, psychotherapist, dietician, district nurse, psychologist, social worker, nurse
3	60	Once every six weeks	8	Family physician, practice nurse, physical therapist, occupational therapist, social worker, district nurse, neighbourhood care

### Data collection

During phases 2 and 3, we used an iterative approach with ongoing data collection from observations, interviews with participants and a focus group meeting. Table 2 offers an overview of the qualitative data collection.

**Table 2:** Qualitative data collection.

Team	Team observations (*n* = 12)	Individual interviews (*n* = 25)	Disciplines interviewed	Focus group meeting (*n* = 6)

1	Round 1	N = 3	Family physician, physical therapist, practice nurse	Practice nurse & physical therapist
	Round 2	N = 2	Practice nurse, occupational therapist	
	Round 3	N = 2	Family physician, practice nurse	
	Round 4	N = 3	Practice nurse (× 2), family physician	
2	Round 1	N = 3	Psychologist, practice nurse, family physician	Dietician & district nurse
	Round 2	N = 2	Physical therapist, district nurse	
	Round 3	N = 2	Practice nurse, district nurse	
	Round 4	N = 2	Occupational therapist, district nurse	
3	Round 1	N = 2	Social worker, physical therapist	Social worker & district nurse
	Round 2	N = 2	Practice nurse, district nurse	
	Round 3	N = 0	No team member was available	
	Round 4	N = 2	Occupational therapist, district nurse	

#### Observations

Access to the meetings was arranged by the teams’ contact persons. Meetings were audio-recorded. A non-participant observer (JvD) took field notes and collected background data using an observation list (Additional file 1). In total 12 meetings were observed (4 per team).

#### Interviews

Participants were selected by means of pragmatic sampling. After each observation, we intended to individually interview at least two other team members. This resulted in 25 interviews. The interview guide (Additional file 2) started with an open question to explore how respondents felt about the meeting that had just taken place. Other questions were related to barriers and facilitators regarding the applicability of the programme, added value and possible improvements of the programme. The interview guide was previously pilot-tested on two members of an IPT, to see how questions were interpreted, and subsequently improved. Interviews lasted on average 15 minutes and were conducted by JvD and a fellow researcher experienced in interviewing.

Next to the individual interviews, also group interviews took place. At the end of each training activity, participating chairpersons briefly evaluated the value and content of the course as a group.

#### Focus group meeting

After the last action research cycle, we organized a focus group meeting (two hours) with representatives from the three participating teams. Teams were asked to delegate two members from different disciplines (other than the chairperson) to represent their team. In total six team members participated. The purpose of this meeting was to share experiences regarding the use of the tools and components of the programme among the three teams, with the aim of providing improvements to the programme. An experienced researcher (MvB) moderated the meeting, while a second researcher (JvD) was responsible for facilitating the meeting and taking notes. During the first hour participants introduced their own work problems and questions. The second hour consisted of sharing experiences regarding the application and added value of the different components of the programme.

### Analysis

Directed content analysis was used to analyse all data collected in phases 2 and 3 [19]. This variant of qualitative content analysis, described by Hsieh and Shannon, combines a deductive and an inductive approach [19]. Within this approach, existing theory and research results are used to develop a coding scheme in advance, to bring more focus in analysing the data [20]. We structured the analysis process with a preparation and organising phase, as described by Elo (2008) [20].

#### Preparation phase

As a first step, a detailed description of each observation was made, based on the observed topics presented in additional file 1, and completed with field notes about significant events and non-verbal communication. The audio-recorded interviews were transcribed verbatim. Hereafter, two researchers (JvD and SB) read all observation reports and transcripts independently and repeatedly in order to familiarize themselves with the data.

#### Organising phase

A preliminary coding scheme, based on earlier findings and literature was developed and used for coding both observations and interviews. This coding scheme included codes related to the five change objectives (Figure 2). Nvivo 10 software was used to store and code all data. After coding the researchers compared their codings and discussed them until consensus was reached [21].

Data that could not be coded using the existing codes were identified and analysed later to determine whether they represented a new category or a subcategory of an existing code. Field notes and the written comments of the researcher were kept and used in the analysis process to enhance the trustworthiness of the study.

### Results

Results are described per phase of action research. First, the development and subsequent content of the draft programme are described (phase 1 of action research), followed by findings from the testing and evaluation phases (phases 2 and 3 of action research). Lastly, the main results of the evaluation and the final version of the programme are presented (phase 4 of action research).

### Developing the draft programme

In addition to the preconditions (Figure 2), the development team had to take into account the dynamic interprofessional team setting. From a theoretical perspective, the Total Process Coaching of Groups (TPCG) model, developed by Goossens, appeared to be a useful framework to obtain a better understanding of group development and team dynamics [22]. The development team agreed to use the TPCG model as a supporting framework, since the model fitted the findings from our needs assessment. The model offers an understanding of complex group processes, from the perspective of their interrelationships, and focuses on the links between four core elements (1) group development; (2) communication levels; (3) leadership; (4) the context of the group. Based on the findings of our needs assessment and the theoretical insights derived from the TPCG model, we assumed that an appropriately trained chairperson might play a significant role in chairing meetings and guiding team development (Figure 2). Based on programmes preconditions (Figure 2), the development team developed a multifaceted programme mainly intended to improve the functioning of IPTs by facilitating and training chairpersons. Table 3 offers background information on the components of the draft programme.

**Table 3:** Components of the draft programme and suggested adjustments.

Components of the draft programme	Description	Facilitators (+) and barriers (–) regarding the draft programme	Suggested adjustments

**Team reflection framework**	Periodic reflection offers teams the opportunity to share experiences and issues, and eventually improve functioning. The draft programme included the instruction to periodically reflect on team functioning during team meetings, based on the levels of communication as described within the TPCG model (content, procedures, interaction, personal, context). During reflection, the chairperson was supposed to ask stimulating questions, guide the evaluation and group analysis, to eventually draw conclusions and set learning objectives.	+ Awareness of own performance + Active role of the chairperson + Secure group climate – Socially desirable answers – Little input – Superficial reactions	Form Periodically scheduling in time for reflection
			Content Clear questions for reflection In-depth reflection Using the shared rules as starting point
**Team instruction meeting**	Kick-off meeting (1 hour) to inform and motivate all team members.	+ Informative + All team members involved – Too much information in a short time – Time-consuming	Form Add Instruction videos Manageable background information package
**Chairperson training**	Training course focused on organizing and structuring IPT meetings, monitoring the patient perspective and *guiding the team through development* (including managing team dynamics and group processes). As part of the training course, the programme also included two peer feedback sessions to learn from and with each other.	+ Structure + Focus on the patient + Attention to group processes and team dynamics + Time investment and workload + Cross-pollination: Learn from and about each other – Insufficiently context-specific – One session is insufficient – Lack of peer assessment in the workplace	Form Additional training session More variety in form Peer feedback and consultation Add on-the-job coaching
			Content More customizing training content More attention for reflection Emphasizing the core values (patient-centredness) Training chairpersons explicitly to adopt a directive style of leadership and act like a leader who anticipates group dynamics Train chairpersons to become change agents Add video material
**Format for getting to know each other**	As a first step to improve team functioning, the draft programme offers a format that facilitates getting to know each other as a team.	+ Positive team climate + Most of the team members know each other (by name, discipline) – Lack of knowledge of each other’s specialty and competences	Form Add a ‘face book’
			Content Have a round of introductions Gain insight into each other’s frame of reference by introducing everyone’s personal and organizational contexts
**Format to discuss ground rules**	A format with topics which can be used by the team to discuss and capture shared rules and agreements.	+ Shared rules lead to greater clarity and uniformity + Useful for new team members + Efficiency + Increases mutual respect – Time-consuming – Some concepts are confusing – Conflicting views among team members – Too many rules – Some participants show little interest, fail to see the benefit	Form Draft rules prepared by chairperson and discussed by the rest of the team Actively involving team members during development of the draft rules in the start-up phase Attractive design Chairperson’s role to ensure follow-up
			Content Simplified terminology
**Format to structure interprofessional team meetings**	The interprofessional meeting structure provides a framework comprising a three-phase structure (preparation, meeting itself, follow-up) that can support teams in conducting efficient meetings.	+ Clear and satisfactory meeting structure + Tight scheduling resulting in more efficiency and time being saved + Something to hold on to and structure the meeting – Risk of losing the strength of the old (less structured) approach	Content Possibility to introduce patient cases ad hoc
**Overview of organizational roles (chairperson, secretary, introducer)**	Overview of the tasks and responsibilities of four organizational roles that can be distinguished during IPT meetings. Roles include: chairperson, minutes secretary, presenter (of the patient’s case), and participant.	+ Clear expectations + Division of workload + Creates a sense of fellowship + Interplay between chairperson and secretary + Chairperson acts as driving force and leader + Raising awareness of contributor’s role – Double role as chairperson and presenter – Lack of directive leadership – Not everyone is playing a role or contributes to the meeting – Presenters experience difficulties in presenting patient cases	Form Permanent secretary
			Content Appointing and training a second chairperson Expectations of membership Adopting a directive style of leadership More time to practice presenting patient cases
**Form for preparing the meeting**	A form which can be used to support discussing the patients. This form should be completed by the person presenting the case, and sent to the other team members prior to the meeting. The form includes the following components: name and discipline of the presenter, reason for presenting the patient case, description of the patient’s situation, stating the problem. The form also includes a description of the patient’s functioning and goals in a variety of domains related to patient’s health status (somatic and cognitive), activities and participation, environment (physical and social), the way the patient self manages and the resulting care agreements.	+ Targeted preparation – Time consuming – Threshold to filling in for – Terminology – Unclear instructions – A lot of paperwork	Form Better accessible and user friendly Conveniently sized version
			Content Adjustment of terminology Simplify Possibility to introduce patient cases ad hoc
**Six-step plan to discuss the patient in a patient-centred way**	The plan contains six steps to discuss patient’s care plans in a patient-centred way. (1) Describing the patient’s situation, (2) goals and motivation, (3) analysis, (4) brainstorming on possible actions, (5) formulating concrete care agreements, (6) evaluation.	+ Patient-centredness – Inclined to skip steps – Too detailed – Unaware of the steps	Form Simplifying Conveniently sized version (placemat)
			Content Simplified

### Testing and evaluating the draft programme

Three teams used the draft programme. They started with the team instruction meeting, and experimented with the tools. The composition of the participating teams slightly differed per meeting and could therefore be perceived as flexible. The teams’ chairpersons attended the training activities. In order to analyse the results, we describe the findings per change objective. For each programme component, the facilitators, barriers and suggested adjustments in terms of format and content are described and presented in Table 3.

#### 1. Knowing each other

Both observations and interviews showed that most of the team members knew their colleagues by name or face. However, due to changes in team composition over time, and the fact that some members were only occasionally present, teams frequently had to deal with new team members. This caused situations in which team members were not familiar with each other’s expertise, impeding collaboration. As a possible strategy, participants suggested to add a proposal round each time a new team member joins. Others recommended the use of a “face book” with some professional and personal details of all team members plus a photo:

“There are some people whom you don’t know as well as others, and contacts with those people are less smooth.” (Physical therapist, practice 1)

#### 2. Clear structure and organization

Participants recommended that teams should agree on the goal of the meeting, the language used, content of the meeting, organization (frequency, time span, location), structure of the meeting, division of roles, exchange of information, presentation of cases, and reflection on and evaluation of meetings. Participants explicitly mentioned that for them, division and specification of organizational roles created clarity. Participants recommended documenting the agreements made, so they can be easily handed to new team members. According to participants, the drafting of and adherence to these rules and arrangements can be considered a shared investment. Some mentioned that the rules were not properly applied in practice due to the strong and deviant views of some of their colleagues, confusing concepts, or the fact that some members had not read the rules. As a solution, participants emphasized the importance of discussing and formulating the rules jointly with the team:

“It’s good that there is now a concrete agreement. That puts pressure on people. You have to stick to this now. Before, … if it was different each time, then it didn’t matter … this offers more guidelines. Everyone now comes prepared, which wasn’t always the case before.” (Practice nurse, practice 1)

Observations showed that the three-phase IPT meeting structure (Table 3) as introduced by the chairperson was adopted by the teams in a natural way. The follow-up part, including feedback to the patient, was evaluated particularly favourably by participants. Summarizing the action points at the end of the meeting yielded clearly defined results. Participants also valued that the meetings were tightly scheduled, resulting in more efficiency:

“In my view, the main advantage is that everything is more tightly planned. You can now schedule in your next patient appointment precisely. It used to be that you had to schedule out another hour, as you never knew whether it was going to end at 2 or at 3. So it’s better from a practical point of view.” (Physical therapist, practice 3)

However, some participants questioned the potential risk of working in a too structured manner, which might mean losing some of the advantages of an informal approach. They mentioned the necessity of maintaining the possibility to discuss acute situations ad hoc. Lastly, participants believed that becoming acquainted with the meeting structure can be considered a growth process, in which the team has yet to find its way, which takes time.

#### 3. Patient-centredness

The form used to prepare and present patients supported participants in making up a complete picture of the patient (Table 3). Some participants filled in the complete form, while others used key words or only used parts of it. Most mentioned that using the form stimulated them to prepare the meeting in a targeted way, which resulted in time gain during the meeting and increasing patient-centredness. However, some reported to struggle with formulating and presenting realistic and achievable patient goals and concerns, which was visible during the observations. They argued that filling in the form is a labour-intensive activity that could take too much time. This could hamper just quickly discussing an acute patient, and be a barrier to presenting new patients. Observations showed that the number of patients discussed had decreased since the form was introduced:

“It definitely takes a lot of work. And that prevents you from just quickly presenting a patient’s case. You have to write down a whole history. Just spontaneously presenting a case on the spot is out of the question.” (District nurse, practice 2)

As a result, participants contended that the possibility to discuss patients with unexpected and urgent problems ad hoc, without using the form should be maintained. They further agreed on a flexible way of completing the form, in which the different parts should be described succinctly. Despite the suggestion of simplifying the way the form has to be completed, participants also suggested various topics to be added: feedback to the patient, informed consent from the patient, case manager or ultimately responsible person, question of the presenter, and current care and currently involved disciplines. Moreover, participants appreciated the possibility to tailor the form to their own specific needs. To safeguard patients’ privacy, one of the practices experimented with discussing patients anonymously. However, this was perceived by some of the team members as unsatisfactory, making them feel unfamiliar and disconnected with the patients discussed.

Further, participants appreciated following the six-step approach since it was clearly defined and provided the necessary structure to appropriately discuss patients. The form used to prepare the meeting and the six-step plan were seen as mutually strengthening aspects. However, observations showed that teams struggled to stick to the six steps in the suggested way, resulting in care agreements that were not always specific. Interviewees acknowledged that the steps were not yet fully integrated into their work routine. They were not used to talking about patients’ personal goals, and tended to define these goals themselves, from their professional perspective:

“It’s not part our routine, I think, to think on the basis of the patient’s goals. You do discuss it with them, but to explicitly define them… I guess that wasn’t part of our education.” (Practice nurse, practice 1)

Other participants mentioned that detailed completion of all six steps took too much time, making people skip some of the steps. Others indicated that the draft plan was too detailed, and should be simplified into a conveniently sized format like a “place-mat”. Participants also considered that keeping track of all the steps and ensuring that the other team members are included in the discussion were primary tasks of the chairperson. In meetings where the steps were followed systematically, the chairperson was visibly monitoring the steps by naming them and summarizing the results after each step:

“It’s the chair’s task to ensure that the six-step plan is followed. It’s not up to me to keep track of that and check what I have to do now and what is the next step. I’m very willing to follow the chair’s lead in that. He’s the one who has to ensure that we stick to the plan.” (Family physician, practice 1)

#### 4. Feedback and team reflectiveness

Most participants considered team reflection as valuable for progress. They perceived the reflection framework to be useful in guiding team reflection. However, observations showed that reflection was frequently conducted in general terms. An opening sentence often used by the chairperson was:

…“What did we all think of the team meeting? And what about using the new guidelines … How did you experience that?” (Practice nurse, practice 3)

Some mentioned lack of time for reflection or reported negative experiences with reflection. They stated that it was difficult to talk openly in front of the team, as it made them feel vulnerable. Others regarded reflection as superficial, yielding socially desirable answers, or were critical about compulsory reflection. Observations indicated that only a minority of the team members were actively participating. Further, some participants believed that reflection was not always needed, and expected that if the team is functioning well, there will be no need to reflect regularly. Participants mentioned that the use of reflection was still in its early stages, and teams were still searching for a way to include reflection in their current routines. One of the challenges of reflection is to get everyone to express their opinions and to avoid ending up with superficial reactions. As a possible point for improvement, participants mentioned the importance of involving all team members, especially the unmotivated:

“I don’t think anybody is feeling, like, I’m afraid to say what I feel. There’s always room for reflection.” (Psychologist, practice 2)

In addition, participants stated that a secure group climate, characterized by security and trust is needed to reflect freely about group atmosphere. Getting to know each other and team building were mentioned as relevant for creating a secure climate. In order to enable reflection, participants proposed to schedule it as integral part on the agenda. Further, to avoid superficial reactions, participants suggested that reflection should be more specific and proposed using video recordings of own meetings:

“If you just ask is everything OK, they’ll just say fine. But if you say ‘What about this specific issue, does anybody have any comments on that?’ … then they’ll say Oh yes, actually. You have to have a starting point for reflection.” (Family physician, practice 1)

#### 5. Chairing meetings and guiding team development

Findings emphasize the essential role of the team’s chairperson in organizing and structuring IPT meetings, and guiding the team development. Most participants preferred a chairperson who was not afraid to take the lead by activating and motivating team members. Given the continuously changing composition of teams, participants recommended to train chairpersons specifically to adopt principles of a directive style of leadership, and to correctly anticipate group dynamics. The chairperson should play an active role by intervening on content, procedure and interactional level. During reflection, the chairperson ought to ask stimulating questions, guide the evaluation and group analysis, to eventually draw conclusions and set learning objectives. Regardless of the participants’ preference for a more directive chairperson, they mentioned that involving the team in the decision-making process, and letting team members think along is crucial in creating a sense of belonging:

“The chairperson is really the leader. We’re a kind of fellow-travellers who tag along. At least that’s how I perceive it. She really takes up that role wholeheartedly. And it works very well.” (Occupational therapist, practice 3)

Participating chairpersons experienced both the training course and the peer feedback as valuable and learned a lot from each other. However, they regarded a 4-hour training as too short, and recommended expansion with a second session. In addition, participants expressed a desire for direct feedback and on-the-job coaching. Some chairpersons perceived their role as leader to be complex, especially when they had to fulfil the double role of chairperson and presenter of a patient case. As possible solution for support and peer feedback they recommended to appoint and train a second chairperson in each team as a co-chair. Lastly, the participating chairpersons highlighted the importance of tailoring the content of the training to the specific team context and chairpersons’ learning objectives.

### Final programme

**Figure 3:**
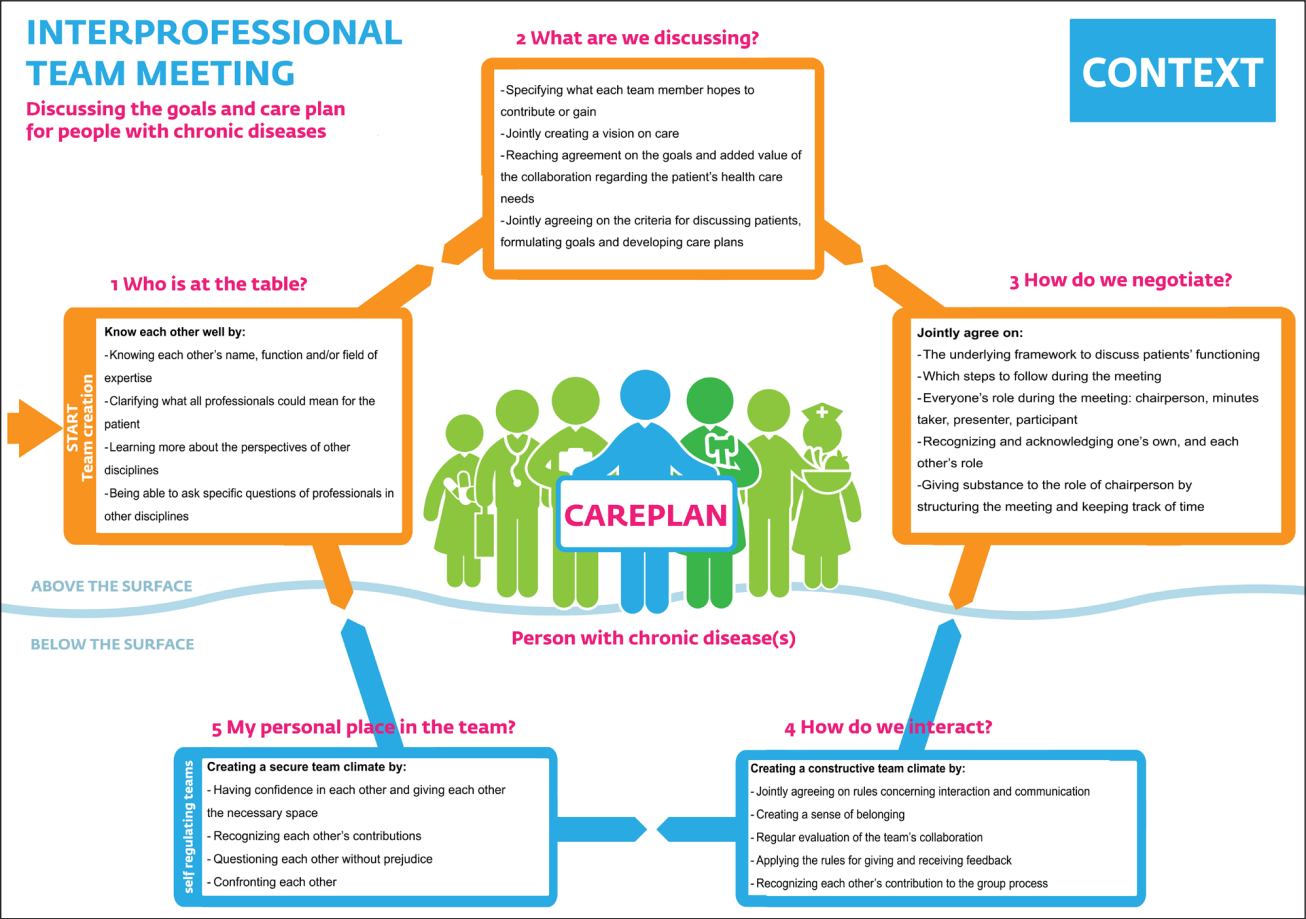
Framework to reflect on interprofessional team functioning.

Based on the findings of phases 2 and 3, we adjusted both the form and content of the draft programme (Table 3). The training course was expanded by an additional session, and on-the-job coaching (comprises observing the chairperson during a meeting and providing direct feedback and feedforward immediately afterwards). A second team member is also to be trained as a co-chair and sparring partner. Tools were adjusted based on the participants’ suggestions (Table 3), and expanded with supplementary video materials and brochures. The programme’s core content now comprises a multifaceted framework (based on the TPCG model) that can be used by interprofessional primary care teams to reflect on the various dimensions and functioning of their meetings (Figure 3). Team reflection enables the programme to be customized and tailored to a team’s specific context and learning objectives. In the final programme (**Box 2**), the training course includes two sessions to develop leadership skills, and skills needed to organize and structure meetings, ensure patient-centredness and guide the team development.

**Box 2: Final programme**

**Reflection framework**

– Framework to reflect on team functioning (Figure 3)

**Training activities** (*for chairperson and co-chair*)

– Team instruction meeting
– Two training sessions
– Two peer feedback sessions
– On-the-job coaching

**Toolbox**

– Format for getting to know each other
– Format to discuss ground rules
– Format to structure interprofessional team meetings
– Overview of organizational roles
– Form to prepare meetings
– Six-step plan to discuss patients
– Video (instruction) material
– Brochure

## Discussion

This article describes the lessons learnt from the systematic, iterative development process of a programme to improve structure, patient-centredness and leadership of interprofessional team (IPT) meetings, using an action research approach. The final programme comprises a reflection framework for interprofessional primary care teams, a team instruction meeting, training for the persons chairing such meetings, as well as a toolbox.

Knowing the team members personally and building up a relationship of trust and respect can be regarded as a precondition for achieving a sustainable collaboration [13]. Knowing each other personally enables professionals to take advantage of each other’s discipline-specific competences. Our findings show that getting to know each other remains an ongoing concern, in view of the continuously changing team composition in primary care.

Further, the process of jointly coming to agreements and setting group rules for the content, procedure, and the interaction of meetings was appreciated by participants, contributing to a positive team spirit. Our findings show that after general agreement has been reached on the structure of the meetings, the team offers support and the chairperson is able to initiate the new structure easily. Similarly, other studies have shown that roles and responsibilities in such meetings need to be clearly articulated and negotiated [23,24]. Although most participants supported a structured approach, they also appreciated the positive effect of viewing the meetings as a social gathering with an informal approach.

For achieving patient-centredness, the draft programme included a form that can be used to put a patient’s case on the agenda of the meeting, and present it at the meeting. Most participants valued the form, although some experienced its use as time-consuming and a possible barrier to presenting patients, preventing them from discussing patients ad hoc. In addition, a six-step approach was developed to structure the way the patients are discussed. Observations showed that professionals tended to quickly move from describing the patient’s situation to proposing possible solutions, reflecting their apparent eagerness to carry out interventions and provide care. The professionals reported skipping the step of defining patients’ goals because this is not part of their culture and work routine. However, a qualitative study of patients’ perspectives of IPT meetings has shown that patients do expect health care professionals to put them at the centre and follow a structured as well as holistic approach to address their needs [25]. Often, patients also appreciate having a voice in their own care process and having the opportunity to attend or be represented during team meetings [17,25]. However, it is not common practice for professionals to invite patients to take part in IPT meetings [13,14]. Some professionals mention it is time consuming to involve patients, and others fear that they will lose some freedom of speech [26,27]. Nevertheless, inviting them or their representatives to take part could be considered a possible way to ensure patient-centredness.

Moreover, participants regarded improving their teams’ functioning by adopting new structures as a growth process. They considered periodic team reflection as a possible approach to improvement. However, team reflection is not self-evident. During the time set aside for reflection, the chairperson ought to ask stimulating questions, guide the evaluation and joint analysis, and eventually draw conclusions and set learning objectives. Our reflection framework (Figure 3) appeared a useful aid for participants to reflect on their functioning.

Lastly, the role of the chairperson as a “change agent” appeared even more important than anticipated. In addition to the technical aspects of structuring meetings and monitoring the agreed rules, leadership appeared crucial. It is known that active participation by leaders increases the chances of successful implementation of quality improvement projects [28]. From a theoretical point of view, and according to the TPCG model, it appears preferable to think of leadership in terms of situational leadership. In this approach, originally developed by Hersey and Blanchard, the style of leadership depends on the group’s current circumstances and specific situation [22]. However, given the continuously changing composition of interprofessional primary care teams, a directive style of leadership appeared to be preferable. This leadership style is characterized by a leader who initiates ideas and tasks, and sets out a clear course [22], and who is able to make decisions and to empower the other team members to collaborate [23,29]. Research directed at IPT meetings in stroke rehabilitation, also found that this directive leadership style is associated with effective meetings, resulting in clear decisions [30]. In addition to the directive style, participants of our study recommended to also adopt principles of the coaching style. Coaching is known to be positively associated with the level of team members’ satisfaction with the team, team climate and positive emotions [31]. Our participants recommended supporting the chairperson by appointing and training a second person who should function as co-chair and sparring partner. The interplay between the two chairpersons, characterized by sharing responsibilities and complementing each other’s area of expertise, can be regarded as co-leadership, contributing to the provision of sustainable integrated care [32]. In addition to the training course they had been provided with, our chairpersons expressed a need for direct feedback and coaching, and accordingly suggested to add on-the-job coaching as a component to the training course.

During the development process, we became more aware of the influence of contextual preconditions on the way IPT meetings are conducted. Such contextual preconditions include the nature, size and composition of the team, how the team has existed, the location, but also external conditions like financial systems and laws. It becomes clear that effective development of interprofessional teams requires support from the wider organizational context [33]. Contextual issues were also highlighted in another study which concluded that impacts of interventions designed to enhance interprofessional teamwork vary under the influence of professional and organizational contexts [23]. Flexibility in terms of local adaptation and customizing appears desirable. Ideally, the content of the training course and the application of both framework and toolbox should be tailored to the context and specific setting of a participating team. A study on coaching interprofessional teams confirms this finding and concluded that since there is no “one size fits all” solution, it is essential to explore and adjust to the team’s specific context [34].

### Strengths and limitations

The strengths of this study are the cyclic process in which research, action and evaluation were interlinked and intended users were continuously involved. This was achieved by leaving the decision making to the development team in collaboration with the three participating interprofessional teams, making the findings more likely to be incorporated into practice [35]. The trainers (JD and WG) served as coaches to the teams, rather than trying to persuade them with their own insights. This increased the team members’ receptivity to change, and encouraged them to take action themselves. Another strength was the practice of discussing issues in the team, rather than between individuals outside the team [36]. The empirical evidence for the TPCG model, used as underlying theoretical framework is limited. Although this can be considered a potential weakness, the model fits in with existing theories and proved to be useful and practically feasible for the participants. The participating teams volunteered, were intrinsically motivated, and recognized the importance of efficient and patient-centred team meetings. In this respect, however, they may not be representative of average primary care teams, which could be a potential source of bias. For practical reasons, the individual interviews were rather short (15 minutes). This might have caused us to miss some in-depth findings. However, by interviewing a range of professionals from different professional backgrounds, we included many perspectives. Another strength was the use of data triangulation by using different sources to check for inconsistencies and divergent perspectives across the data set. Initial analyses were undertaken by one researcher and checked by a second researcher to ensure consistency of coding and safeguard against selective use of data. After four cycles of action research we concluded to have reached data saturation, since we did not extract any new insights from the last cycle.

## Conclusion

This article reports on the systematic development of a programme to improve IPT meetings in primary care. The programme comprises a multifaceted framework that can be used by interprofessional primary care teams to reflect on the various dimensions and functioning of their IPT meetings. Findings revealed the important role of the chairperson as change agent initiating reflection and guiding the team through development. We developed a training course for two members per team, focussing on the effective organization and structuring of IPT meetings and safeguarding the patient’s perspective. The programme also included a toolbox to assist teams in improving their effectiveness and efficiency. Getting acquainted with new structures can be considered a growth process, in which teams have to find their way. To be effective, the programme should be customizable and adapted to the teams’ specific contexts and dynamics. Further research is needed to determine the actual value and manageability of the programme.

## Additional Files

The additional files for this article can be found as follows:

- ijic-18-1-3076-s1.pdf
  **Additional file 1** Semi-structured interview guide. DOI: https://doi.org/10.5334/ijic.3076.s1
- ijic-18-1-3076-s2.pdf
  **Additional file 2** Observation guide. DOI: https://doi.org/10.5334/ijic.3076.s2
