# Supplementary material for: Development of a Customizable Programme for Improving Interprofessional Team Meetings: An Action Research Approach
Source: Int J Integr Care. 2018 Jan 25;18(1):8. doi: 10.5334/ijic.3076 (PMC5887069; doi:10.5334/ijic.3076)
Supplement: Supplementary file 1 [file ijic-18-1-3076-s1.pdf]

Additional file 1: Semi-structured interview guide

Interview guide

- Introduction
  - Introduction of the interviewer
  - Consent for audio recording
  - Explanation of participants' rights
  - Aim of the interview: The aim is to explore personal experiences (both barriers and facilitators) regarding the new way of working and the use of the new tools and methods.
  - Procedure, duration etc.
  - Emphasizing that all information is relevant

Introduction question: *What did you think of the interprofessional team meeting which has just taken place?*

Subquestions:

- *Can you give an example?*
- *Can you explain in detail what you mean by…?*
- *Why do you perceive ...as a relevant factor/barrier/facilitator?*

Main questions:

Questions concern the use, experience, handling, added value and suggestions for improvement of the different components of the programme.

1. *To what extent do the team members know each other?*
   a. What do you think of this?
   b. How would you suggest to improve this?
2. *To what extent did the meeting go according to the agreed rules for the meetings?*
   a. How were the rules applied according to you?
   b. How did you personally feel about handling these rules?
   c. What are barriers and facilitators regarding the application of these rules?
   d. What do you need to apply these rules?
   e. In your view, did working according to these rules provide added value?
   f. Did you miss any topics on which agreements and rules have to be made?
   g. Do you have any suggestions for improvement?
3. *Which roles were fulfilled during the team meeting? What was your role? (Chairperson, secretary, presenter, participant)*
   a. How did feel about fulfilling your role?
   b. What do you need in order to fulfil your role properly? (What factors influence the fulfilment of roles?)
   c. In your view, did the division of roles offer added value? And why?
   d. Do you have any suggestions to improve the way roles are fulfilled?
4. *What can you tell us about working according to the new meeting structure?*
   a. What did you think about working according to the new meeting structure?
   b. What parts of the structure did or didn't you adhere to? And why?
   c. What did you perceive as barriers to working according to the new meeting structure? What factors influence this?
   d. In your view, did working according to this new meeting structure offer added value? And why?
   e. Do you have any suggestions for adjustment and improvement of the meeting structure?
5. *How did you prepare for the discussion of a patient?*
   a. What did you think of preparing the meeting?
   b. To what extent did you use the form to prepare your presentation of a patient?
   c. What can you tell us about the way you used the form?
   d. Were there parts of this form that you found difficult to fill in? If yes, why?
   e. What helped you to prepare the meeting properly? (What factors influence this?)
   f. In your view, did preparing your presentation of the patient case using this form offer added value? And why?
   g. How would you suggest the preparation of the meeting could be further improved?
6. *How did you discuss patients?*
   a. What did you think of the use of the six-step plan to discuss patients?
   b. What steps did you go through? (And if you skipped certain steps, why did you do so?)
   c. What factors influence the process of following the different steps?

   d. *In your view, did the use of this six-step plan to discuss patients offer added value?*
   e. *Do you have any suggestions to improve the six-step plan?*

7. *Did you evaluate and reflect together? If yes, what did you think of this and what can you tell us about it?*
   a. On which topics did you reflect/ evaluate? (content, procedure, interaction)
   b. What does a team need in order to make reflection possible?
   c. In your view, did periodic reflection and evaluation offer added value?
   d. Do you have any suggestions to improve the team reflection effort?
8. *How was it to feed back the suggested agreements to the patient?*
   a. What bottlenecks did you experience?
   b. How did the patient react?
9. *To what extent was the patient's perspective (goals and wishes) focused on during the meeting? Could you give an example?*
   a. Do you have any suggestions to improve the patient-centredness of interprofessional team meetings?

<u>Summary:</u>

Interviewer gives a summary.

- *Did I forget any relevant information in this summary?*
