# Supplementary material for: Development of a Customizable Programme for Improving Interprofessional Team Meetings: An Action Research Approach
Source: Int J Integr Care. 2018 Jan 25;18(1):8. doi: 10.5334/ijic.3076 (PMC5887069; doi:10.5334/ijic.3076)
Supplement: Supplementary file 2 [file ijic-18-1-3076-s2.pdf]

Additional file 2: Observation guide

**Observation list**<br><br>**Overall**	**Is the programme (core component / tool) being applied, and if so, which parts?**<br>*How (and to what extent) are the different components and tools of the programme being applied?*	**Manageability and feasibility**<br>*What do you notice about the feasibility of the programme?*<br>**Bottlenecks**<br>*What are the evident bottlenecks?*<br>**Contextual factors**<br>*Which visible contextual factors are influencing the application of the programme?*<br>**Added value**<br>*What can you say about the added value provided by the programme?*
Knowing each other	○ Team members know each other (No one has to be introduced, no new faces)	
Rules for the meeting	○ Everyone visibly adheres to the agreements and rules for the meeting<br>○ Team members stick to the organizational agreements	
Roles	○ **<u>Chairperson:</u>**<br>○ Has prepared the meeting<br>○ Structures the meeting<br>○ Ensures adherence to the rules of the meeting<br>○ Follows the six-step plan<br>○ Summarizes<br>○ Keeps track of time<br>○ Guides reflection<br>○ Points out tensions<br>○ Clearly announces closing the meeting and plans next meeting<br><br>○ **<u>Secretary:</u>**<br>○ Takes care of the reporting (preparing minutes)<br>○ Checks regularly whether minutes correspond to what has been agreed on during discussion<br>○ Where appropriate, refers to previous minutes	
Interprofessional meeting structure	○ **<u>Preparation</u>**<br>○ Clear agenda<br>○ Information about patients is provided before the meeting<br><br>○ **<u>Meeting</u>**<br>○ Opening<br>○ Discussion of each patient<br>○ Closing<br><br>○ **<u>Aftercare</u>**<br>○ Reporting<br>○ Agreements regarding feedback to patient<br>○ Agenda and agreements for next meeting	
Interaction and team climate	○ **<u>Team members give each other feedback</u>**<br>○ On content<br>○ On procedure<br>○ On interaction<br><br>**Any comments about the team climate and atmosphere?**<br>**Are there any visible irritations?**<br>**Any comments about the way the team talks about patients?**	
Reflection tool	**<u>At which level does the team reflect?</u>**<br><br>○ Is there any input from participants?<br>○ Are specific learning objectives defined?<br>○ Does the team refer back to previous agreements and learning objectives?	

**Observation list**<br><br>**Per patient**	**Is the programme (core component / tool) being applied, and if so, what parts?**<br>*How (and to what extent) are the different parts and tools of the programme applied?*	**Manageability and feasibility**<br>*What can you say about the feasibility of the programme?*<br>**Bottlenecks**<br>*What are the evident bottlenecks?*<br>**Contextual factors**<br>*Which visible contextual factors are influencing the application of the programme?*<br>**Added value**<br>*What can you say about the added value provided by the programme?*
Roles	**Presenter:**<br>○ Gives a clear description of the patient's background and situation<br>○ Presents the patient's personal goals and wishes<br>○ Presents an explicit question for the team<br>○ Follows the six-step plan<br><br>**Participants:**<br>○ Active participation<br>○ Clearly prepared (do not ask questions that are redundant)	
Preparation	○ Presenter has filled in the form correctly and completely (there are no questions or uncertainties)<br>○ The form has been submitted on time and according to the agreed rules	
Six-step plan	○ Description of the patient's situation<br>○ Patient's goals and motivation<br>○ Analysis<br>○ Formulating concrete proposals for action<br>○ Formulating concrete care agreements<br>○ Evaluation	
